# Factors predicting technical failure of endoscopic transpapillary gallbladder drainage for acute cholecystitis

**DOI:** 10.1002/deo2.308

**Published:** 2023-10-31

**Authors:** Noriyuki Hirakawa, Kenjiro Yamamoto, Atsushi Sofuni, Takayoshi Tsuchiya, Kentaro Ishii, Reina Tanaka, Ryosuke Tonozuka, Shuntaro Mukai, Kazumasa Nagai, Yukitoshi Matsunami, Hiroyuki Kojima, Hirohito Minami, Ryosuke Nakatsubo, Kyoko Asano, Takao Itoi

**Affiliations:** ^1^ Department of Gastroenterology and Hepatology Tokyo Medical University Tokyo Japan

**Keywords:** cholecystitis, endoscopic gallbladder stenting, endoscopic nasogallbladder drainage, gallbladder, transpapillary gallbladder drainage

## Abstract

**Objectives:**

Endoscopic transpapillary gallbladder drainage (ETGBD) is a highly technical procedure, but few studies have evaluated factors that predict its technical success. Therefore, in this study, we sought to identify predictors of technically successful ETGBD.

**Methods:**

One hundred and eighty‐two patients who underwent ETGBD for acute cholecystitis at our hospital were retrospectively investigated. Factors associated with technical failure were identified by focusing on clinical characteristics, anatomical features (direction of the cystic duct branch and course of the cystic duct), and procedural factors (cystic duct and gallbladder with or without contrast and cystic duct injury).

**Results:**

The technical success rate was 84.6% (154/182) and the clinical success rate was 96.1% (148/154). The adverse event rate was 11.0% (20/182; cystic duct injury in 13 patients, pancreatitis in six, and liver abscess in one. Univariate and multivariate analyses identified the right cranial direction and spiral‐type course of the cystic duct to be significant anatomical features and cystic duct injury to be a significant procedural feature contributing to the technical failure of ETGBD.

**Conclusions:**

ETGBD is a highly practical procedure for patients with acute cholecystitis. However, difficulty is encountered in some cases because of anatomical and procedural factors. Our results suggest that ETGBD may be difficult and thus should not be performed in cases with the right cranial direction or spiral‐type course of the cystic duct or those with cystic duct injury.

## INTRODUCTION

Laparoscopic cholecystectomy is the gold standard for the treatment of gallbladder disorders, including symptomatic cholelithiasis and acute cholecystitis.^1^ However, for high‐risk surgical patients with organ dysfunction or underlying comorbidities, urgent cholecystectomy is challenging because of significantly increased cholecystectomy‐related morbidity and mortality.^2^, ^3^

The updated 2018 Tokyo guidelines for the management of acute cholecystitis recommend percutaneous gallbladder drainage (PTGBD) as the first‐line alternative to surgery.^4^ However, although PTGBD is an effective treatment for cholecystitis, it is contraindicated in some patients, including those with a bleeding tendency as a result of antiplatelet/anticoagulant therapy or thrombocytopenia and those with massive ascites, and it is also contraindicated when the location is anatomically inaccessible, as in patients with Chilaiditi syndrome.^5^ Furthermore, the external drainage catheter may cause pain or discomfort, which has a negative impact on quality of life, and significant cholecystitis recurrence rates ranging from 22% to 47% have been reported in patients who do not undergo cholecystectomy following removal of the percutaneous catheter.^6^, ^7^

Endoscopic transpapillary gallbladder drainage (ETGBD), which includes endoscopic nasogallbladder drainage (ENGBD) and endoscopic gallbladder stenting, has emerged as an alternative therapy for patients with acute cholecystitis in whom PTGBD is contraindicated.^8^, ^9^, ^10^, ^11^, ^12^, ^13^, ^14^, ^15^ ETGBD has the following benefits over PTGBD: (a) lower risk of bleeding because of the omission of needle puncture; (b) feasibility in patients in whom the anatomical location of the gallbladder is not amenable to PTGBD; (c) simultaneous treatment of common bile duct stones; and (d) no need for an external drainage catheter. ETGBD is clinically effective in resolving symptoms and inflammatory markers but is technically challenging.^16^ ^.^ ^17^ Recent meta‐analyses of ETGBD have shown a pooled technical success rate of 83%,^17^, ^18^ which is lower than that for PTGBD.^19^, ^20^, ^21^ Furthermore, there is a risk of endoscopic retrograde cholangiopancreatography‐related adverse events and perforation of the cystic duct during ETGBD. Essential technical aspects of ETGBD include identifying the cystic duct and cannulation of the cystic duct and gallbladder, and several methods have been reported to improve the technical success of ETGBD.^19^ However, few studies have examined the procedural and anatomical factors affecting the technical success of this procedure. Therefore, in this study, we sought to identify factors that affect the technical success of ETGBD.

## METHODS

### Patients

This retrospective cohort study was performed at Tokyo Medical University Hospital and included patients diagnosed with acute cholecystitis based on clinical symptoms, laboratory data, and imaging studies according to the 2018 Tokyo guidelines between December 2012 and May 2021. The following exclusion criteria were applied: (1) patients in whom the endoscopic approach was difficult because of problems with insertion of the endoscope (e.g., trismus or gastric outlet obstruction), those with surgically altered gastroduodenal or biliary anatomy, and those who were unwilling to participate or unable to provide written informed consent.

The diagnosis and severity of acute cholecystitis were determined according to the 2018 Tokyo guidelines. The patients were initially treated with antibiotics and extracellular fluid infusion and then underwent emergency laparoscopic or open cholecystectomy if eligible. ETGBD was the first choice for patients with Chilaiditi syndrome, massive ascites, coagulation or platelet abnormalities, or complicated common bile duct stones; otherwise, the drainage method was selected on a case‐by‐case basis.

All patients provided written informed consent for the endoscopic procedure. The study was approved by the Medical Ethics Committee of Tokyo Medical University Hospital (approval number T2020‐0029) and was conducted in accordance with the Declaration of Helsinki.

### ETGBD procedure

ETGBD was performed using a standard duodenoscope (JF‐260V, TJF‐Q260V, or TJF‐Q290V; Olympus Medical Systems). After successful cannulation of the bile duct, cholangiography was performed to detect the location of the cystic duct. Next, a 0.032‐inch hydrophilic guidewire (Radifocus; Terumo Co, Ltd.) and cannula (ERCP catheter from MTW Endoskopie Manufaktur; CleverCut3V sphincterotome from Olympus Medical Systems) were used for seeking and advanced into the cystic duct. If the cystic duct was not contrasted, the cystic duct was blindly sought with a guidewire after aspiration of as much bile juice as possible to collapse the bile duct. The guidewire and cannula were then advanced into the cystic duct and a contrast medium was injected to evaluate the shape and bifurcation of the cystic duct. After the guidewire and cannula were advanced into the gallbladder, the gallbladder was aspirated until the infected bile disappeared. The guidewire was then changed to a stiff type (Visi Glide2; Olympus Medical Systems), after which the cannula was removed and the guidewire was placed in the gallbladder. Thereafter, depending on the patient's background, drainage was performed using a 5‐ or 6‐Fr nasobiliary drainage tube with a pigtail shape at the tip of the tube or a 7‐Fr plastic stent with a double‐pigtail shape at both tips of the stent. If ENGBD was performed, the ENGBD tube was cut in the stomach or a gallbladder stent was placed after the inflammation improved. An endoscopist with more than 10 years of experience in endoscopic retrograde cholangiopancreatography performed the procedures. The patients received flunitrazepam for sedation and pentazocine or pethidine for analgesia. If ETGBD was technically or clinically unsuccessful, endoscopic nasobiliary drainage was placed and PTGBD was performed.

### Study outcomes

Data were collected on age, sex, time of onset, inflammation, severity of disease, diameter of the common bile duct, presence or absence of stone formation, shape of the cystic duct, bifurcation of the cystic duct, findings on cholangiography, and cystic duct injury. The primary outcomes were the technical success rate, clinical success rate, and adverse events.

### Definitions

Technical success was defined as the placement of a nasogallbladder drainage tube or a gallbladder drainage stent in the gallbladder. Clinical success was defined as improvement in clinical symptoms and improvement of inflammation on blood tests within 3 days of the procedure. Adverse events were assessed for severity according to the American Society for Gastrointestinal Endoscopy Lexicon grading system.^22^

### Statistical analysis

Patient characteristics and factors potentially associated with technical failure were examined for statistical significance. Continuous variables were transformed into dichotomous variables using the median as the cut‐off value. Multivariate analysis of factors affecting the technical failure of ETGBD was performed using factors with a *p*‐value of <0.2 in the univariate analyses. A *p*‐value of <0.05 was considered statistically significant in multivariate analysis. All statistical analyses were performed using IBM SPSS Statistics software (version 26; IBM Corp.).

## RESULTS

**FIGURE 1:**
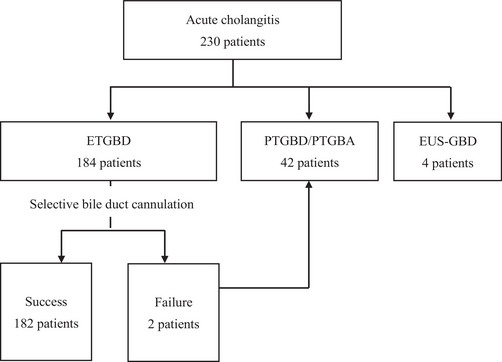
Patient flowchart. ETGBD, endoscopic transpapillary gallbladder drainage; EUS‐GBD, endoscopic ultrasound‐guided gallbladder drainage; PTGBA, percutaneous gallbladder aspiration; PTGBD, percutaneous gallbladder drainage.

**FIGURE 2:**
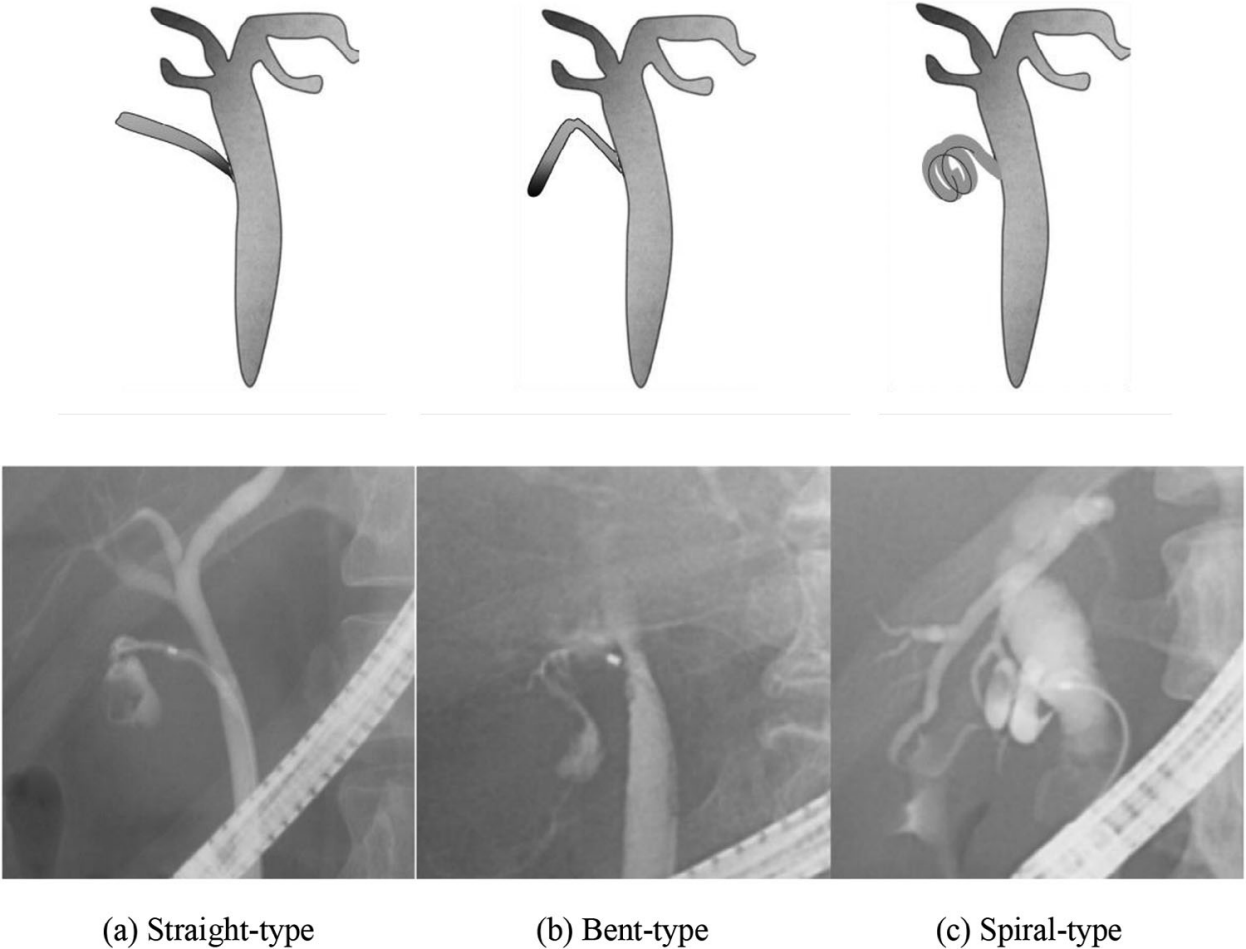
Classification of the course of the cystic duct. (a) Straight‐type, (b) bent‐type, and (c) spiral‐type.

Figure 1 shows the study enrollment process. A total of 230 patients were diagnosed with acute cholecystitis at our hospital during the study period. Forty‐two of these patients underwent PTGBD or percutaneous gallbladder aspiration and four underwent endoscopic ultrasound‐guided gallbladder stenting, leaving 184 patients who underwent ETGBD. After the exclusion of two patients in whom intubation of the bile duct was difficult, 182 patients (117 males, 65 females; median age 74.5 years) who underwent ETGBD were enrolled in the study. Their demographic and clinical characteristics are summarized in Table 1. The number of patients taking antiplatelet/anticoagulant agents was 59. The mean bile duct diameter was 7.0 mm, the mean leukocyte count was 12.8 × 10^3^/μL, the mean C‐reactive protein level was 15.8 mg/dL, and the mean time to intervention was 71.6 h. Cholecystitis was mild in 63 cases, moderate in 80, severe in 26, and unknown in 13. The anatomical features of the cystic duct were classified according to the direction of the cystic duct branch and the course of the duct. The classification of the cystic duct was determined by magnetic resonance cholangiopancreatography (MRCP) and cholangiography, and cholangiography was performed in the prone position. MRCP was referred to in 26 cases. MRCP was performed immediately before the procedure in only one case, and 25 cases had undergone MRCP in the past. In terms of the direction of the cystic duct, a right cranial branch was identified in 127 cases, a right caudal branch in 32, a left cranial branch in 21, and unknown in two. The course of the cystic duct was straight‐type in 50 cases, bent‐type in 105, spiral‐type in 19, and unknown in eight (Figure 2).

**TABLE 1:** Patient characteristics.

	Total
Number of patients	182
Sex, male/female, *n*	117/65
Age, mean ± SD (years)	71.8 ± 13.7
Presence of antiplatelet/anticoagulant agents, *n*	59
Time to intervention, mean ± SD (h)	71.6 ± 55.2
CBD diameter ± SD (mm)	7.0 ± 2.51
WBC count, mean ± SD (× 10^3^)	12.8 ± 5.76
CRP concentration, mean ± SD (mg/dL)	15.8 ± 10.9
GB wall thickness, mean ± SD (mm)	4.9 ± 2.23
Severity of cholecystitis	
Mild	63 (34.6%)
Moderate	80 (43.9%)
Severe	26 (14.2%)
Unknown	13 (7.1%)
Stone location	
Cystic duct stone	58 (31.9%)
Gallbladder stone	63 (34.6%)
Unknown	71 (39.0%)
Cystic duct direction	
Right/cranial	127 (69.8%)
Right/caudal	32 (17.6%)
Left/cranial	21 (11.5%)
Unknown	2 (1.1%)
Course of the cystic duct	
Straight	50 (27.5%)
Bent	105 (57.6%)
Spiral	19 (10.4%)
Unknown	8 (4.4%)

Abbreviations: CBD, common bile duct; CRP, C‐reactive protein; GB, gallbladder; SD, standard deviation; WBC, white blood cell.

ETGBD had a technical success rate of 84.6% (154/182) with a clinical success rate of 81.3% (148/182) in intention‐to‐treat analysis and 96.1% (148/154) in per‐protocol analysis. According to the anatomical characteristics of the cystic duct, the technical success rate was 89.8% (114/127) for a right cranial branch direction, 65.6% (21/32) for a right caudal branch direction, 76.2% (16/21) for a left cranial branch direction, 96.0% (48/50) for straight‐type course of the duct, 89.5% (94/105) for bent‐type, and 63.2% (12/19) for spiral‐type (Table 2).

**TABLE 2:** Anatomy of the cystic duct.

	Total	Success rate	*p*‐value
Direction of the cystic duct			
Right/cranial	127	89.8% (114/127)	0.342
Right/caudal	32	65.6% (21/32)	<0.05
Left/cranial	21	76.2% (16/21)	0.255
Position of the cystic duct			
Proximal	147	84.3% (124/147)	
Distal	35	85.7% (30/35)	0.812
Course of the cystic duct			
Straight	50	96% (48/50)	0.237
Bent	105	89.5% (94/105)	0.189
Spiral	19	63.1% (12/19)	<0.05

**FIGURE 3:**
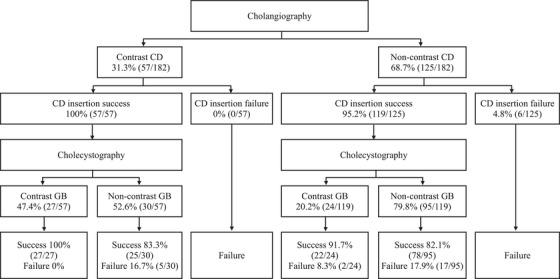
Procedural factors affecting the technical success rate. CD, cystic duct; GB, gallbladder.

In terms of procedural factors, when contrast medium was injected into the common bile duct, the cystic duct was contrasted in 31.3% of cases (57/182) and not in 68.7% (125/182). The technical success rate was 93.0% (53/57) when the cystic duct was contrasted and 81.6% (102/125) when it was not. When contrast medium was injected into the cystic duct, the gallbladder was contrasted in 31.3% of cases (57/182) and not in 68.7% (125/182). When contrast was injected in the cystic duct, the success rate was 95% if the gallbladder was contrasted and 79.8% if not (Figure 3).

Adverse events were observed in 20 of the 182 patients (11.0%): cystic duct injury in 13 (7.1%), mild pancreatitis in five (2.7%), moderate pancreatitis in one (0.6%), and liver abscess in one (0.6%; Table 3).

**TABLE 3:** Adverse events associated with endoscopic transpapillary gallbladder drainage.

Adverse events, *n* (%)	20 (11.0%)
Cystic duct injury	13 (7.1%)
Pancreatitis	6 (3.3%)
Mild	5 (2.7%)
Moderate	1 (0.6%)
Liver abscess	1 (0.6%)

In univariate analysis, cystic duct injury, a right cranial direction, and a spiral‐type course were identified to be significant predictors of technical failure (Table 4). These factors, as well as patient sex and age and a non‐contrasting cystic duct, all of which had a *p*‐value of <0.2 in univariate analysis, were entered into multivariate analysis. Cystic duct injury, right cranial direction, and spiral type were identified by multivariate analysis to be significant predictors of technical failure in ETGBD (Table 5).

**TABLE 4:** Potential predictors of technical failure of endoscopic transpapillary gallbladder drainage identified in univariate analysis.

	Failure	Success	*p*‐value
Sex (male)	19	98	0.669
Age (≥75 years)	13	69	0.683
Presence of antiplatelet/anticoagulant agents	7	52	0.362
Time to intervention (≥3 days)	11	53	0.545
Leukocyte count (≥12.5 × 10^3^/μL)	18	61	0.279
CRP (≥16 mg/dL)	16	68	0.486
Common bile duct diameter (≥7 cm)	19	74	0.635
GB wall thickness (≥5 mm)	14	69	0.555
Branch to GB neck (≥23 mm)	6	60	0.357
Severity grade of cholecystitis (mild/moderate + severe)	8/20	55/86	0.429
Position of the cystic duct (proximal/distal)	22/5	124/32	0.812
Direction of the cystic duct (Right caudal/[Right cranial + Left cranial])	11/18	21/119	<0.001
Course of the cystic duct (spiral/straight + bent)	7/15	12/140	0.006
Cystic duct injury	11	2	<0.001
Non‐contrast cystic duct	23	102	0.095
Non‐contrast gallbladder	28	103	0.017

Abbreviations: CRP, C‐reactive protein; GB, gallbladder.

**TABLE 5:** Predictors of technical failure of endoscopic transpapillary gallbladder drainage identified in multivariate analysis.

	OR (95% CI)	*p*‐value
Sex (male)	0.95 (0.325–2.77)	0.925
Age (≥75 years)	0.925 (0.332–2.57)	0.881
Direction of the cystic duct (right caudal/right cranial + left cranial)	3.81 (1.39–10.4)	0.009
Formation of the cystic duct (spiral/straight + bent)	4.05 (1.08–15.2)	0.038
Cystic duct injury	33.7 (6.31–180)	<0.001
Non‐contrast cystic duct	0.728 (0.20–261)	0.626
Non‐contrast gallbladder	0.384 (0.10–1.56)	0.180

Abbreviations: CI, confidence interval; OR, odds ratio.

## DISCUSSION

In this study, we sought to identify factors that predict the technical success of ETGBD. The procedure had a technical success rate of 84.5% (154/182) and a clinical success rate of 81.3% (148/182) in intention‐to‐treat analysis and 96.1% (148/154) in per‐protocol analysis. Adverse events were observed in 20 (11.0%) of the 182 cases, with cystic duct injury in 13 cases (7.1%), mild pancreatitis in five (2.7%), moderate pancreatitis in one (0.6%), and liver abscess in one (0.6%). These results are in line with previous reports.^16^, ^17^, ^18^ In multivariate analysis, we also identified a right caudal direction and spiral‐type course of the cystic duct and cystic duct injury to be significant predictors of technical failure of ETGBD (Table 5).

Over the years, a number of researchers have investigated the clinical, anatomical, and procedural factors associated with technical failure in ETGBD. In terms of clinical factors, Yane et al identified older age to be a risk factor for failure of ETGBD ^23^ and attributed this finding to the fact that endoscopists are concerned about the risk of procedure‐related adverse events in elderly patients and perform the procedure as quickly as possible. Maruta et al.^24^ identified a dilated common bile duct (≥7.3 mm) to be a predictor of an unsuccessful procedure. The finding might reflect the difficulty of hooking the guidewire in the cystic duct when the common bile duct is dilated. In our study, there was no significant relationship between the technical success rate and the diameter of the bile duct, which might reflect our practice of aspirating bile juice to collapse the bile duct during the procedure.

With regard to anatomical factors, Maruta et al reported that a caudal direction of the cystic duct predicted the technical failure of ETGBD.^24^ Similarly, in our study, the success rate of ETGBD was lower in the right caudal direction than in the right or left cranial direction (Table 2). This finding may be a consequence of the guidewire bouncing upstream of the common bile duct when advancing the guidewire into the gallbladder in a case with a cystic duct in the caudal direction. However, we further validated our results by classifying the cystic duct according to whether its course was straight‐type, bent‐type, or spiral‐type. We found that the success rate was significantly lower for the spiral type than for the other two types. For the spiral‐type cystic duct, adhesions may be present because of inflammation, making it difficult to insert a guidewire into the gallbladder. Furthermore, the spiral‐type cystic duct is also at higher risk of injury than the other types (Table 6). Sato et al reported an association of cystic duct injury with technical failure of ETGBD.^25^ Another report showed that when a cystic duct injury occurs, the guidewire is dislocated from the perforation site into the peritoneal cavity, making it difficult to insert the guidewire into the lumen of the cystic duct in approximately half of cases.^25^

**TABLE 6:** Cystic duct injury according to the type of course of the cystic duct.

	Cystic duct injury	*p*‐value
Straight	4.0% (2/50)	0.311
Bent	7.6% (8/105)	0.771
Spiral	16.6% (3/18)	0.122

In our study, the presence or absence of contrast in the cystic duct was identified in univariate analysis to be a significant procedural factor affecting the success of ETGBD. No contrast in the cystic duct might indicate stone impaction or adhesion as a result of severe inflammation in the duct.

We examined the different aspects of ETGBD failure in this study, but are there useful ways to overcome difficult cases? Yoshida et al reported that the success rate was significantly higher with the use of SpyGlass DS‐assisted ETGBD than with the use of conventional ETGBD.^26^ The SpyGlass DS system was found to be particularly useful when it was difficult to identify the cystic duct and in cases of caudal bifurcation. Intraductal ultrasonography can also be used to identify the cystic duct. Moreover, the SpyGlass DS system can assist correct advancement of the guidewire into a deeper cystic duct because manipulation of the device can occlude the orifice of the cystic duct and prevent the guidewire from going upwards into the proximal common bile duct.^26^ However, in a spiral‐type case, the cystic duct is narrowed by inflammation with adherence or torsion, and insertion may be difficult, even when using a DS. Furthermore, the insertion of a DS may increase the pressure in the bile duct, which may lead to cholangiovenous reflux.

The advent of the lumen‐apposing metal stent (LAMS) has led to endoscopic ultrasound‐guided gallbladder drainage (EUS‐GBD) becoming an attractive non‐surgical gallbladder drainage option.^27^ A meta‐analysis of eight studies that included 393 patients found that EUS‐GBD with LAMS had a cumulative technical success rate of 94.9% and a clinical success rate of 94.6%.^28^ Another meta‐analysis compared EUS‐guided transmural drainage with endoscopic retrograde cholangiography‐assisted transpapillary drainage and found that EUS‐guided transmural drainage resulted in higher technical and clinical success rates (odds ratio [OR] 3.91, 95% confidence interval [CI] 1.52–10.09), *p* = 0.005 and OR 4.59, 95% CI 1.84–11.46, *p* = 0.001, respectively) and a lower recurrence rate (OR 0.17, 95% CI 0.06–0.52, *p* = 0.002).^29^ Therefore, compared with ETGBD, EUS‐GBD has superior efficacy and a lower risk of recurrence. However, the use of LAMS for gallbladder drainage remains off‐label, and EUS‐GBD can only be performed by skilled pancreatobiliary endoscopists in a limited number of facilities. Furthermore, some concerns remain regarding EUS‐GBD, including post‐procedure cholecystectomy and the long‐term results of permanent LAMS placement.^30^, ^31^ Nevertheless, ETGBD has distinct advantages over EUS‐GBD, including physiological drainage that preserves the native anatomy for future surgical intervention and favorable long‐term endoscopic gallbladder stent placement.^32^, ^33^ In addition, with the aging of the population, the number of patients on antithrombotic drugs is expected to increase and ETGBD will be required more often in the future.

This study has some limitations It had a single‐center retrospective design and a small sample size, which might result in selection bias and low external validity. Nevertheless, this study is one of the few to examine the technical aspects of ETGBD in detail.

In conclusion, ETGBD is a highly practical procedure for patients with acute cholecystitis. However, difficulties may be encountered in some cases because of clinical, anatomical, and procedural factors. Therefore, ETGBD should only be performed after a thorough understanding of the case has been obtained by preoperative and intraoperative contrast imaging. Our results suggest that ETGBD may be difficult and thus should not be performed in cases with the right cranial direction or spiral‐type course of the cystic duct or those with cystic duct injury.

## CONFLICT OF INTEREST STATEMENT

Takao Itoi is the Editor‐in‐Chief of DEN Open. Takao Itoi and Takayoshi Tsuchiya are consultants for Olympus. The rest of the authors declare no conflict of interest.
